# Antiviral Effect of Amentoflavone Against Influenza Viruses

**DOI:** 10.3390/ijms252212426

**Published:** 2024-11-19

**Authors:** Won-Kyung Cho, Hee-Jeong Choi, Syed Sayeed Ahmad, Inho Choi, Jin Yeul Ma

**Affiliations:** 1 Korean Medicine (KM) Application Center, Korea Institute of Oriental Medicine (KIOM), 70 Cheomdan-ro, Dong-gu, Daegu 41062, Republic of Korea; chj1901@kiom.re.kr; 2 Department of Medical Biotechnology, Yeungnam University, Gyeongsan 38541, Republic of Korea; sayeedahmad4@gmail.com (S.S.A.); inhochoi@ynu.ac.kr (I.C.); 3 Research Institute of Cell Culture, Yeungnam University, Gyeongsan 38541, Republic of Korea

**Keywords:** amentoflavone, anti-influenza viral effect, hemagglutinin, neuraminidase, virucidal effect

## Abstract

Amentoflavone (AF) is a biflavonoid compound found in many plants. In this study, we first demonstrate that AF has a potent antiviral effect against the influenza virus via the inhibition of viral attachment and virucidal effects. The anti-influenza-viral effect of AF was evaluated using green fluorescent protein-tagged Influenza A virus (IAV) with fluorescent microscopy and flow cytometry analysis. AF decreased the GFP expression by viral infection, dose-dependently. Fifty micromoles of AF suppressed the GFP expression by virus infection of up to 70% of untreated infected control cells. Consistently, immunofluorescence results showed the inhibitory effect of AF on viral protein expression. Time-of-addition and hemagglutination assays revealed that AF inhibits viral binding to cells by interfering with the hemagglutinin (HA) of IAV. Furthermore, AF has a virucidal effect and blocks cytopathic effects caused by the Influenza B virus and H3N2 IAV. Additionally, AF represses the neuraminidase (NA) activity of IAV. In silico analysis confirmed the potential interaction of AF with both HA and NA. Our findings indicate that AF has antiviral effects by modulating HA and NA during the attachment and release stages of influenza viral infection.

## 1. Introduction

Annually, there are 3–5 million cases of epidemic influenza and up to 650,000 people die from seasonal influenza-related respiratory disease worldwide [1]. Influenza viruses are in the Orthomyxoviridae family and are classified into A, B, and C [2]. In particular, the Influenza A virus (IAV) is highly contagious and spreads rapidly, resulting in epidemics or pandemics [2]. IAV is categorized into subtypes based on the antigenic properties of two surface glycoproteins, hemagglutinin (HA) and neuraminidase (NA) [3]. Influenza A virus is an RNA virus and has 12 proteins including matrix proteins 1, 2 (M1, M2), nucleoproteins (NPs), nonstructural proteins (NS), and RNA polymerase. HA and NA of Influenza A have opposite functions related to interaction with sialic acid, which is a receptor for the virus [4]. HA is responsible for viral binding to the receptor containing sialic acid and NA is critical for the progeny release by cleaving the sialic acid of the membrane. Zanamivir and oseltamivir, which are NA inhibitors, have been developed and used as IAV drugs. However, side effects of and drug resistance to NA inhibitors have been reported [5,6,7]. Currently, baloxavir, targeting RNA polymerase A of IAV, is in clinical trials for efficacy and safety [8,9,10]. Since IAV frequently mutates with a high error rate due to RNA-dependent polymerase, it is impossible to develop the perfect vaccine and antiviral drug to confront IAV [11,12,13]. In this regard, plant-based extracts and bioactive compounds with safety and efficacy are good candidates as novel antiviral agents against the influenza virus.

Amentoflavone (AF) is a well-known polyphenolic compound in many plants, including calophyllaceae [14,15]. According to reported studies, AF exhibits various biological activities such as anti-inflammatory, neuroprotective, antioxidative, anti-atopic, antitumor, and antiviral activity [16,17,18]. In particular, AF showed antiviral effects against several viruses such as HIV, herpes simplex virus type 1, and hepatitis B and C virus [19,20,21]. Aoki-Utsubo, Chie et al. demonstrated that AF suppresses hepatitis B virus (HBV) infection through the inhibition of HBV attachment, implying the potential antiviral activity of AF against HBV [22]. Lin Y.M. et al. reported that AF exerts an inhibitory effect against IAV, but did not reveal the relevant mechanism for its antiviral activity [23]. In previous research, we found that AF from *Thuja orientalis* exerts the strongest antiviral effect among several active compounds including quercetin, myricetin, quercitrin, and afzelin. However, it is still unknown how AF inhibits IAV infection [24].

In this report, we demonstrate that AF has an antiviral effect against influenza viruses by inhibiting HA and viral binding to the cells and attenuating NA activity, suggesting that AF has a therapeutic effect against influenza viral infection.

## 2. Results

### 2.1. Cytotoxicity of AF

**Figure 1:**
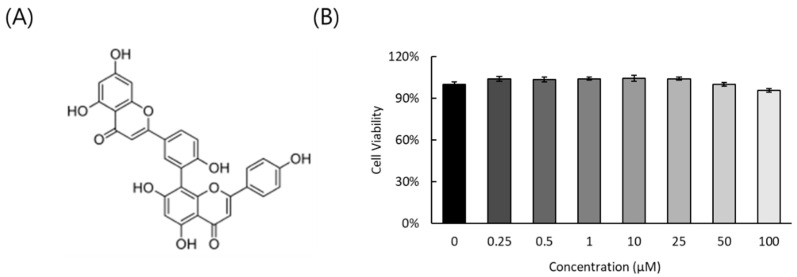
The structure (**A**) and cytotoxicity (**B**) of AF on RAW 264.7 cells. The data represent the mean ± SD based on three replicates in three different experiments.

The cytotoxicity of AF in RAW 264.7 cells was checked using CCK-8 assay. As shown in Figure 1, AF did not show a significant cytotoxic effect up to 100 μM.

### 2.2. AF Inhibits Influenza A Virus Infection, Dose-Dependently

**Figure 2:**
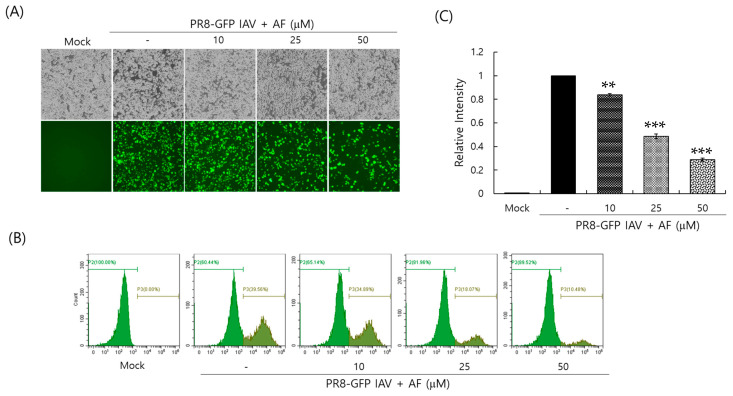
AF inhibits Influenza A viral infection. AF (10, 25, or 50 µM) or medium (mock) was preincubated with PR8-GFP IAV at 4 °C, and the mixture was added to the RAW 264.7 cells at 37 °C for 24 h. (**A**) Brightfield and fluorescent images were captured using fluorescent microscopy (200× magnification). (**B**,**C**) The GFP-expressing cells were counted using FACS. The relative GFP intensities were calculated by comparing them with the PR8-GFP IAV-infected control group. The data represent the mean ± SD based on three independent experiments. Statistical significance was assessed via an unpaired Student *t*-test. *** *p* < 0.0005 and ** *p* < 0.005 compared with virus-infected group. P2: population 2. P3: population 3 of the subgroup.

To investigate whether AF could affect influenza virus infection, we cotreated RAW 264.7 cells with AF at various concentrations and GFP-expressing PR8 Influenza A virus (PR8-GFP IAV). Although MDCK or A549 cells are usually used for influenza viral infection experiments, we used RAW 264.7 cells for IAV infection in this experiment because they are also easily infected by IAV and their small cell size allows for easy relative comparison in concentration-dependent experiments. AF (0, 10, 25, or 50 µM) and PR8-GFP IAV were mixed at 4 °C for 1 h, before infection of the cells. The mixtures were added to the cells for 2 h at 37 °C. The cells were washed to remove the remaining virus and AF and incubated for 24 h. The anti-influenza-viral effect of AF was examined by detecting the GFP expression level of the cells. As presented in Figure 2A, when infected with PR8-GFP IAV, strong GFP expression was observed in control cells. However, GFP expression in the presence of 25 or 50 µM AF was significantly reduced, dose-dependently. We confirmed the inhibitory effect of AF on PR8-GFP IAV infection using FACS analysis. The cells that were uninfected, infected with PR8-GFP virus only, or infected with PR8-GFP and AF at in indicated concentration (10, 25, or 50 µM) were fixed, and GFP-expressing cells were counted by detecting the fluorescein isothiocyanate (FITC) level (Figure 2B). FITC is known to have excitation and emission spectrum peak wavelengths of approximately 495 nm and 519 nm, giving it a green color. The relative GFP expression level of each group was graphed by comparing it with the PR8-GFP virus-only group (Figure 2C). AF inhibited GFP expression with a 50% effective concentration (EC_50_) of 23.01 ± 0.07 µM. Consistent with Figure 2A, AF potently repressed GFP expression by PR8-GFP viral infection. We also confirmed the anti-IAV effect of AF in A549 and MDCK cells. AF was nontoxic in both cells up to 100 μM (Supplementary Figure S1). AF dose-dependently inhibited GFP expression by PR8-GFP IAV infection in both cell groups (Supplementary Figure S2). These results indicate that AF has a strong antiviral effect on the PR8-GFP influenza virus.

### 2.3. AF Represses Influenza Viral Protein Expression

**Figure 3:**
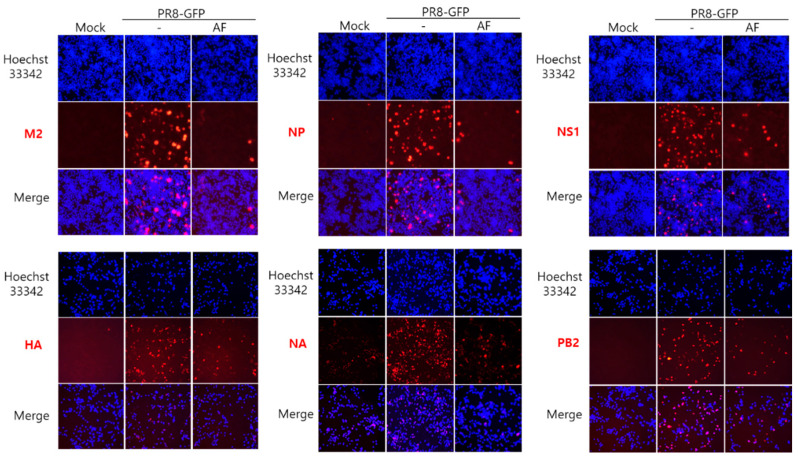
AF represses influenza viral protein expression. AF (25 µM) or medium (mock) was mixed with PR8-GFP IAV and added to the cells. After incubation at 37 °C for 24 h, the cells were fixed and stained with specific antibodies against influenza viral proteins (Red). The nuclei were detected with Hoechst 33342 (Blue). The images were merged for the co-localization of viral proteins and nuclei (200× magnification).

Next, we conducted an immunofluorescence assay to assess the effect of AF on influenza viral protein expression. The cells infected by PR8-GFP IAV in the presence or absence of AF were fixed and stained with Alexa-594-conjugated antibodies specific to IAV proteins. Figure 3 shows that AF significantly decreases the expression of IAV proteins including M2, NP, NS1, HA, NA, and PB2.

### 2.4. AF Dose-Dependently Blocks IAV Infection at the Viral Attachment Step and Exhibits a Virucidal Effect

**Figure 4:**
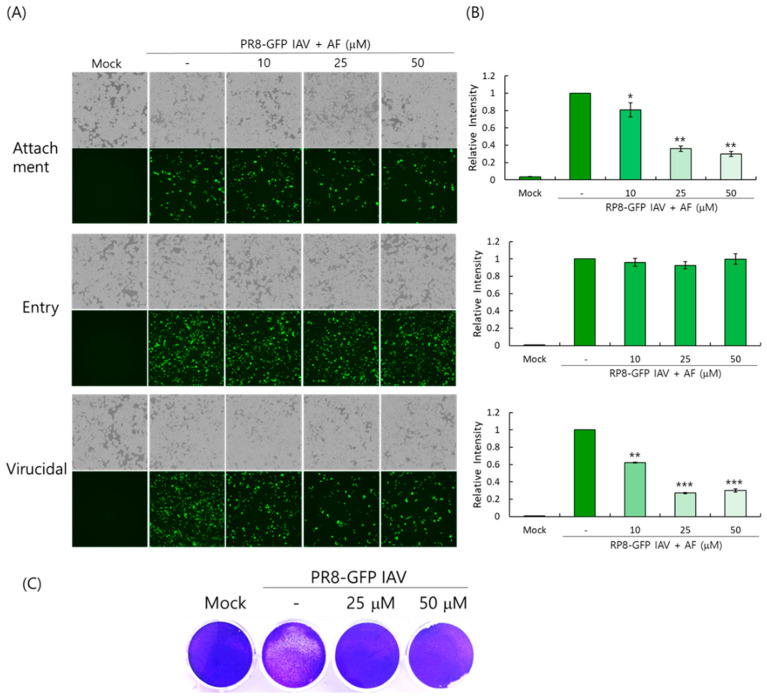
AF significantly inhibits IAV attachment and exerts a virucidal effect on cells before entry. (**A**,**B**) The virucidal effect of AF and its inhibitory effect on attachment and entry were examined using a time-of-addition assay as described in the Materials and Methods. The GFP-expressing cells were captured using fluorescent microscopy (**A**) and FACS analysis (**B**). The data represent the mean ± SD based on three independent experiments. Statistical significance was assessed via an unpaired Student *t*-test. *** *p* < 0.0005, ** *p* < 0.005, and * *p* < 0.05 compared with the virus-infected group. (**C**) AF (10 or 50 µM) and PR8-GFP IAV were incubated at RT for 1 h. The mixtures were added to the MDCK cells at 37 °C for 1 h. After washing, the cells were overlaid with DMEM containing 1.5% agarose and further incubated for 72 h. The cells were fixed and stained with 1% crystal violet.

We performed the time-of-addition assay to determine whether AF could affect viral attachment and entry and induce virucidal effects as described in the Materials and Methods. The experiments for virus attachment, entry, and virucidal effect analysis were performed under different incubation conditions of the virus, AF, and cells at 4 °C. Since viruses only attach to the cell surface at 4 °C and cannot penetrate, the incubation of viruses and AF with cells at 4 °C for 30 min allows us to simultaneously examine the inhibition of the attachment step. The incubation of viruses and cells at 4 °C for 30 min first, followed by washing them with PBS and adding AF, can allow us to examine the inhibition of virus entry into cells. The incubation of viruses and AF first at 4 °C for 30 min can allow us to examine the virucidal effect. Figure 4A,B show that AF dose-dependently inhibits viral attachment, not the entry step. AF at 25 µM reduced viral binding to the cells by up to 60% compared with virus-infected control cells. In addition, AF exerted a strong virucidal effect at 25 µM. We next confirmed the virucidal effect of AF using a plaque inhibition assay. If AF has virucidal activity, the incubation of the virus with AF at room temperature will destroy the virus, reducing the number of viruses in the plaque essay. To address this, AF and PR8-GFP IAV were co-incubated at room temperature for 1 h and the mixture was added to the MDCK cells at 37 °C for 1 h. After washing the remaining viruses, the cells overlaid with DMEM medium containing 1.5% agarose were further incubated for 72 h. As shown in Figure 4C, in the presence of AF, the plaque formation by PR8-GFP IAV was significantly inhibited. These findings suggest that the anti-IAV effect of AF is related to the blockage of AF on viral binding to the cell during infection and the direct virus-destroying effect.

### 2.5. AF Inhibits Hemagglutinin and Neuraminidase of IAV

**Figure 5:**
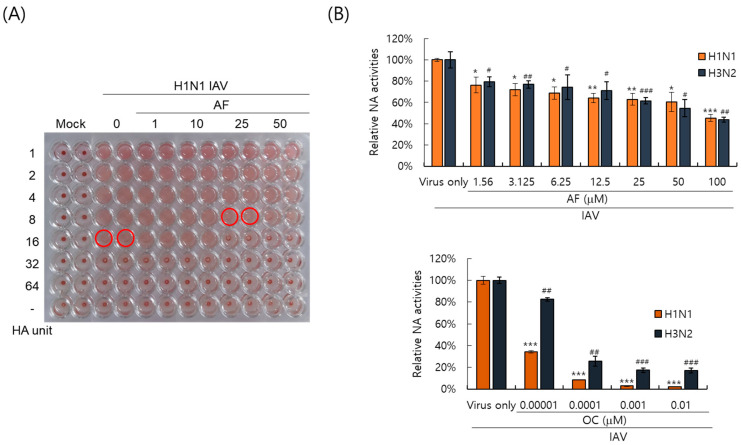
The inhibitory effects of AF on hemagglutination (**A**) and neuraminidase (**B**) activity of Influenza A virus. (**A**) AF at the indicated concentrations or medium (mock) was preincubated with H1N1 IAV for 1 h at 4 °C. The mixture was cotreated to RAW 264.7 cells. The supernatants of the cells were serially diluted and mixed with RBCs. The red circle indicates HA units. (**B**) AF or oseltamivir carboxylate was serially diluted and mixed with H1N1 or H3N2 IAV. An NA inhibition assay was performed according to the manufacturer’s instructions. NA activities were detected using a fluorescence spectrometer. The data represent the mean ± SD based on three independent experiments. Statistical significance was assessed via an unpaired Student *t*-test. *** *p* <0.0005, ** *p* < 0.005, and * *p* < 0.05 compared with the H1N1-virus infected group. ^#^ *p* < 0.0005, ^##^ *p* < 0.005 and ^###^ *p* < 0.05 compared with the H3N2 virus-infected group.

Since hemagglutinin of IAV plays a key role in viral attachment to the cells, we investigated whether AF could affect hemagglutination by IAV using red blood cells. As presented in Figure 5A, IAV in the absence of AF showed 16 HA units; however, in the presence of AF, it was 8 units. AF lowered the HA units of IAV. This result confirms that AF restricts IAV infection by inhibiting virus attachment to cells, consistent with Figure 4. Neuraminidase activity is essential for progeny release and a target for antiviral drugs. We investigated the effect of AF on NA activity. As presented in Figure 5B, AF exhibited an NA-inhibitory effect against both H1N1 and H3N2 IAV from 1.56 μM. In particular, 100 μM of AF suppressed NA up to 50% compared to the virus-infected control. These results imply that AF suppresses both HA and NA of influenza viruses.

### 2.6. AF Suppresses Cytopathic Effect by H3N2 IAV and IBV

**Figure 6:**
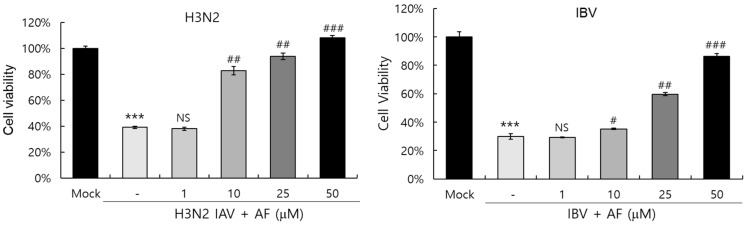
AF blocks the cytopathic effects of H3N2 IAV and IBV dose-dependently. H3N2 IAV or IBV was preincubated with AF (0, 1, 10, 25, 50 µM), and the mixtures were added to RAW 264.7 cells until cytopathic effects were seen. The cell viability was determined by the CCK-8 reagent. The data represent the mean ± SD based on three independent experiments. Statistical significance was assessed via an unpaired Student *t*-test. *** *p* < 0.0005 compared with the mock control, and ^#^ *p* < 0.0005, ^##^ *p* < 0.005 and ^###^ *p* < 0.05 compared with the virus-infected control. NS; no significance.

Next, we examined whether AF could affect cytopathic effects by Influenza A and B viruses. The cell viability assay showed that in the absence of AF, H3N2 IAV and Influenza B virus (IBV) infections decreased viable cells by up to 40% and 20%, respectively, by inducing cytopathic effects in the cells. However, AF potently blocked cytopathic effects by IBV, as well as H3N2 IAV, dose-dependently. In particular, 50 μM of AF increased the cell viability by up to 90% and 100% compared to the non-infected mock control (Figure 6). These findings suggest that AF has a strong protective effect against both IAV and IBV infections.

### 2.7. In Silico Analysis Shows AF Could Interact with Hemagglutinin and Neuraminidase of IAV

**Figure 7:**
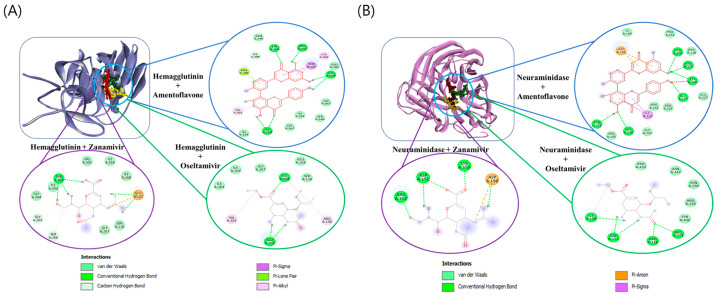
The docking interaction of selected compounds and hemagglutinin (**A**) or neuraminidase (**B**). Three-dimensional representation of amentoflavone (red color), oseltamivir (yellow color), and zanamivir (green color) interactions with hemagglutinin or neuraminidase.

In this study, we investigated the potential for hemagglutinin to interact with oseltamivir, zanamivir, and AF through in silico analyses. The free energy of binding for the complexes such as hemagglutinin + AF, hemagglutinin + oseltamivir, and hemagglutinin + zanamivir was found to be −6.33, −3.18, and −2.25 kcal/mol, respectively. The interacting complex of AF, oseltamivir, and zanamivir with hemagglutinin is shown in Figure 7A. AF showed a better affinity with hemagglutinin than oseltamivir or zanamivr. For the other selected complexes, such as neuraminidase + AF, neuraminidase + oseltamivir, and neuraminidase + zanamivir, the free energy of binding was found to be −3.90, −2.21, and −1.04 kcal/mol, respectively. The interacting complex of AF, oseltamivir, and zanamivir with neuraminidase is shown in Figure 7B. The total number of amino acid residues and H-bond interactions is listed in Supplementary Table S1 for these interactions. The binding affinity of AF with neuraminidase was found to be higher than that of oseltamivir and zanamivir; however, the binding site of AF was found to interact with more amino acid residues near the active site of neuraminidase, likely due to the larger structure of AF than other control compounds. These results suggest that amentoflavone could bind to both hemagglutinin and neuraminidase.

## 3. Discussion

Influenza virus is a respiratory infectious agent that causes massive damage to global health. Although many attempts have been made to combat viral infections, a perfect antiviral drug against influenza virus infection has not yet been developed. In a previous report, we found that amentoflavone in *Thuja orientalis folium* affects influenza virus infection [24]. This research was performed to examine how amentoflavone inhibits influenza viral infection. A CCK-8 assay demonstrated that AF has no cytotoxic effect up to 100 µM in the cells (Figure 1B). When we examined the effect of AF on GFP-tagged influenza virus infection in RAW 264.7 cells, we found that AF significantly represses GFP expression by viral infection, dose-dependently (Figure 2). Consistently, immunofluorescence analysis showed that AF suppresses the expression of IAV proteins including M2, NP, NS1, HA, NA, and PB2 (Figure 3). When we performed the time-of-addition assay to examine which stages are affected by AF, we found that AF inhibits viral binding to the cells at the early stage of infection (Figure 4). Since HA is a critical protein responsible for viral attachment to cells, several HA-inhibitory compounds including aryl sulfonamide [25], oleanolic acid derivatives [26], and stilbene piceatannol [27] are reported as anti-influenza-viral candidates. We also recently reported that a phytochemical, isoquercitrin [28], and herbal extracts including *Hoveniae Semen Seu Fructus* [29] have anti-influenza-viral efficacy via modulating hemagglutinin and inhibiting viral attachment to cells. When we conducted an HA inhibition assay using chicken red blood cells to investigate whether AF could affect the HA of IAV, we found that AF lowered the HA units by 2-fold compared to the virus-infected control, indicating that AF interferes with HA, thereby inhibiting viral attachment to cells at an early stage during infection (Figure 5A). Additionally, AF exhibited a strong virus-destroying effect before attaching to the cells (Figure 4). Its virucidal action may be related to its HA-inhibitory effect, and its ability to bind directly to the virus and consequently inhibit the attachment of virus particles to cells [30]. Luganini et al. reported that the interaction of HA and cranberry extract results in HA interference and viral infectivity loss [31]. Further, AF inhibited the NA of H1N1 and H3N2 IAV, which is essential for viral progeny release. AF at a concentration of 1.5 µM reduced NA activity by 20% compared to virus-infected controls (Figure 5B). AF at 100 µM repressed the activity by up to 50% compared to the control. These results imply that AF blocks IAV at both early and late stages during infection. We examined the antiviral effect of AF against H3N2 IAV and Influenza B virus by evaluating the inhibitory effect on cytopathic effect formation. AF significantly increased cell viability by reducing cytopathic effects due to viral propagation, dose-dependently (Figure 6). AF at 50 µM completely blocked CPE induced by H3N2 IAV and Influenza B virus infection. Collectively, AF exerts antiviral efficacy against various types of influenza viruses such as H1N1, H3N2 IAV, and IBV. In silico analysis indicates that AF could interact with both hemagglutinin and neuraminidase with high affinity (Figure 7), thereby modulating influenza virus infection. Although in vivo further studies need to be conducted, our findings indicate that AF has the potential to be developed as an antiviral drug to protect against IAV infection.

## 4. Materials and Methods

### 4.1. Cells and Viruses

RAW 264.7 (Mouse Leukemic Monocyte Macrophage cell line; ATCC TIB-71) and MDCK (Madin-Darby canine kidney; ATCC NBL-2) cells were grown in Le Roswell Park Memorial Institute medium (Hyclone, Logan, UT, USA) and Minimal Essential Medium (Hyclone, Logan, UT, USA), respectively, with 10% fetal bovine serum and 100 U/mL of Penicillin and Streptomycin at 37 °C with 5% CO_2_. Green fluorescent protein (GFP)-tagged Influenza A (A/PR8/34(H1N1))-GFP and A/PR8/34 (H1N1) viruses were kindly gifted from Dr. Jong-Soo Lee (Chungnam National University, Daejeon, Republic of Korea), and HBPV-VR-32 (H3N2) was purchased from the Korea Bank for Pathogenic Viruses (KBPV). The viruses were propagated in the allantoic fluid of a 10-day-old chicken embryo.

### 4.2. Cytotoxicity Test

Amentoflavone (AF) was purchased from Chemface (Wuhan, China). Powdered AF was dissolved in DMSO. The cytotoxicity of AF was examined with cell counting Kit-8 (CCK-8) according to the manufacturer’s recommendation (Dojindo, Rockville, MD, USA). Briefly, AF was diluted from 0.25 to 100 µM and added to the cells. After 24 h incubation, 10 μL of CCK-8 reagent was added to the cells and incubated for 2 h. Absorbance at 450 nm was measured using a spectrophotometer (Promega, Madison, WI, USA).

### 4.3. Antiviral Activity Against Influenza Virus

Influenza viruses (H1N1-GFP, H1N1, H3N2 IAV, or IBV) were mixed with AF and incubated for 1 h at 4 °C, and the mixtures were cotreated with RAW 264.7 cells for 2 h at 37 °C. After washing with PBS, the cells were further incubated for 24 h until GFP expression or cytopathic effect (CPE) formation was seen. The EC50 of AF on IAV infection was calculated using an online tool: MLA-“Quest Graph™ IC50 Calculator” by AAT Bioquest Inc. (Pleasanton, CA, USA), https://www.aatbio.com/tools/ic50-calculator (accessed on 16 April 2024). A time-of-addition assay was performed to determine the inhibitory step of AF. For the attachment stage, the cells were infected with PR8-GFP IAV (10 MOI) and AF at 4 °C for 30 min. After washing, the cells were incubated at 37 °C for 24 h. For the entry stage, the cells were infected with the virus at 4 °C for 30 min, followed by treatment with AF at 37 °C for 30 min. After washing, the cells were incubated at 37 °C for 24 h. For the virucidal stage, the virus and AF were mixed at 4 °C for 30 min. After washing, the cells were incubated at 37 °C for 24 h.

### 4.4. Fluorescence-Activated Cell Sorting (FACS) Analysis

AF at various concentrations was cotreated with PR8-GFP IAV in RAW 264.7 cells, as described in Section 2.3. The cells were fixed with 4% paraformaldehyde for 10 min and GFP expression using FITC (green color) was detected using a CytoPLEX flow cell counter (Beckman Coulter Inc., Pasadena, CA, USA).

### 4.5. Plaque Inhibition Assay

A plaque inhibition assay was conducted as described in a previous report [29]. Briefly, MDCK cells seeded in 12-well plates at a density of 5 × 10^5^ cells were cotreated with PR8-GFP IAV and AF at 4 °C for 1 h. The mixture of virus and AF was added to the cells at 37 °C for 1 h. After washing with PBS, the cells were overlaid with agar overlay medium (DMEM with 1.5% agarose, 10% BSA) and incubated at 37 °C for 72 h. After fixing with 4% paraformaldehyde for 10 min, the media and agar overlay were removed and the cells were stained with 1% crystal violet.

### 4.6. Immunofluorescence Staining

H1N1 IAV (10 MOI) and 25 µM AF were preincubated for 1 h at 4 °C and the mixture was cotreated in RAW 264.7 cells for 2 h at 37 °C. After washing with PBS, the cells were further incubated for 24 h. The cells were washed with PBS, and then fixed with methanol for 10 min and 4% paraformaldehyde for 10 min. After washing, the cells were blocked with 5% BSA-PBST for 30 min and incubated with specific antibodies against influenza viral proteins for 1 h at room temperature. The cells were incubated with an Alexa Fluor 594-conjugated secondary antibody for 1 h in the dark and incubated with Hoechst 33342 for 5 min in the dark. Proteins (red) and nuclei (blue) were visualized using a fluorescence microscope.

### 4.7. Hemagglutination (HA) Assay

H1N1 IAV (10 MOI) and AF (0, 1, 10, 25, or 50 µM) were mixed and incubated for 1 h at 4 °C. The mixtures were added to the RAW 264.7 cells for 2 h at 37 °C and washed with PBS. After a further 24 h incubation, the supernatants were used for a hemagglutination assay. Each supernatant was serially two-fold diluted and added to a round-bottomed 96-well plate. Then, 1% chicken RBCs (Innovative Research, Inc., Southfield, MI, USA) in PBS were mixed with the samples. After incubation for 1 h at room temperature, the plates were photographed.

### 4.8. Neuraminidase (NA) Inhibition Assay

The neuraminidase (NA) inhibition assay was conducted using the NA-Fluor influenza Neuraminidase Assay Kit (Life Technologies, Carlsbad, CA, USA), according to the manufacturer’s instructions. Briefly, AF was two-fold serially diluted with assay buffer from 100 μM to 1.56 μM and added to a black 96-well plate. As a positive control, oseltamivir carboxylate, a specific NA inhibitor, was used. H1N1 IAV in assay buffer was added to each well and incubated for 30 min at 37 °C. Each sample was incubated with 200 µM NA-Fluor Substrate in a 96-well plate for 1 h for 37 °C and the reaction was terminated by NA-Fluor stop solution. The reaction was detected by a fluorescence spectrometer (Promega, Madison, WI, USA) with an excitation at 365 nm and an emission at 445 nm.

### 4.9. Molecular Docking Analysis

The 3D structures of viral proteins such as hemagglutinin (PDB ID: 1RU7) and neuraminidase (PDB ID: 3TI5) were obtained from RCSB-PDB (www.rcsb.org). The PDB file of these selected targets was cleaned by removing the heteroatoms. Further, the selected positive control (oseltamivir and zanamivir) and test compound (Amentoflavone) were retrieved from the PubChem database (https://pubmed.ncbi.nlm.nih.gov/, accessed on 16 April 2024). Autodock 4.2 [32] was performed to check the interaction between selected compounds and the target. The receptor and ligand preparation were conducted by a docking protocol, and a 60 × 60 × 60 grid box was plotted based on selected interaction sites. Docking results in dlg format were analyzed for the binding energies (kcal/mol) of the interaction between compounds and targets.

## 5. Conclusions

Amentoflavone (AF) exhibits a potent anti-influenza-viral effect. AF inhibits viral binding to cells by interfering with the HA of the influenza virus in the early stage of infection. AF inhibits the release of IAV progeny by targeting NA at a late stage. Our results suggest that AF exhibits a strong antiviral effect against influenza by affecting viral binding and progeny release, and by direct virucidal effects throughout the infection. AF has the potential to be developed into an anti-influenza-viral agent.

## Data Availability

Data is contained within the article.

## Supplementary Materials

The following supporting information can be downloaded at: https://www.mdpi.com/article/10.3390/ijms252212426/s1.
