# Supplementary material for: Antiviral Effect of Amentoflavone Against Influenza Viruses
Source: Int J Mol Sci. 2024 Nov 19;25(22):12426. doi: 10.3390/ijms252212426 (PMC11595079; doi:10.3390/ijms252212426)
Supplement: Supplementary file 1 [file ijms-25-12426-s001.zip › ijms-3305305-supplementary.pdf]

Supplementary data

Sup. Figure S1.

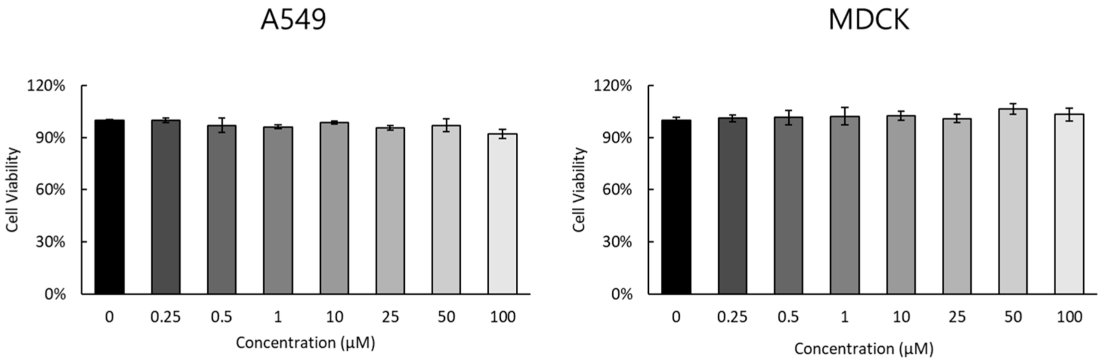

Cytotoxicity of AF on A549 and MDCK cells. AF at the indicated concentrations was treated in the cells for 24 h. The cell viability was determined using CCK-8 assay. The data represent the mean ± SD based on three replicates in three different experiments.

Sup. Figure S2.

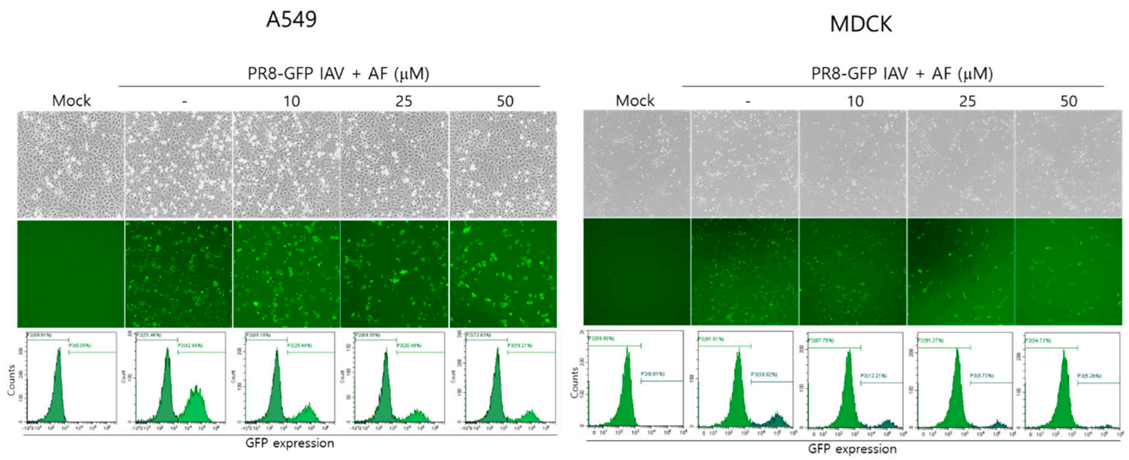

The inhibitory effect of AF against PR8-GFP IAV infection in A549 and MDCK 549 cells. AF (10, 25, or 50 μg/ml) or medium (mock) mixed with PR8-GFP IAV for 1 h at 4°C were cotreated to the cells for 2 h at 37°C. After washing with PBS, the cells were further incubated for 24 h. The levels of GFP expression were evaluated with fluorescent microscopy and FACS analysis.

Sup. Table S1.

The total number of amino acid residues and H-bond interactions present in the complex formation of selected target and compounds

**Target name**	**Compound name**	**H-bond interaction**	**Total number of amino acid residues**
Hemagglutinin	Amentoflavone	ILE268:HN - UNK0:O<br>TYR309:HN - UNK0:O<br>UNK0:H - GLY304:O<br>UNK0:H - PRO307:O<br>UNK0:H - ILE268:O	Gly265, Ser266, Gly267, Ile268, Gln296, Pro300, Val301, Thr302, Gly304, Glu305, Cys306, Pro307, Lys308, Tyr309
	Oseltamivir	ILE268:HN - UNK0:O<br>UNK0:H -GLU107:OE2	Glu103, Arg106, Glu107, Ser110, Gly267, Ile268, Ile269, Ile303
	Zanamivir	THR302:HN - UNK0:O<br>UNK0:H - A:THR302:O<br>UNK0:H - GLU107:OE2<br>UNK0:H - GLU107:OE1	Glu107, Ser110, Gly265, Ser266, Ile268, Ile269, Val301, Thr302, Ile303, Gly304
Neuraminidase	Amentoflavone	ARG292:HH11 -UNK0:O<br>ARG371:HH21 - UNK0:O<br>NUNK0:H - GLU277:OE2<br>UNK0:H - TYR406:OH<br>UNK0:H - ASN221:OD1	Arg118, Ile149, Asp151, Arg152, Asn221, Ile222, Arg224, Glu227, Gly244, Pro245, Ser246, Glu277, Arg292, Arg371, Tyr406
	Oseltamivir	ARG118:HH11 - UNK0:O<br>ARG371:HH11 - UNK0:O<br>UNK0:H - ASP151:OD1<br>UNK0:H - ASP151:OD2	Arg118, Ile149, Asp151, Arg292, Asn294, Asn347, Arg371, Tyr406, Pro431
	Zanamivir	LYS150:HZ2 - UNK0:O<br>ASP151:HN - UNK0:O<br>ARG152:HH11 - UNK0:O<br>UNK0:H - ASP198:OD2	Lys150, Asp151, Arg152, Asp198
